# Direct measurement of topological interactions in polymers under shear using neutron spin echo spectroscopy

**DOI:** 10.1038/s41598-019-39437-2

**Published:** 2019-02-26

**Authors:** Maciej Kawecki, Franz A. Adlmann, Philipp Gutfreund, Peter Falus, David Uhrig, Sudipta Gupta, Bela Farago, Piotr Zolnierczuk, Malcom Cochran, Max Wolff

**Affiliations:** 1 Department of Physics and Astronomy, Uppsala University, P.O. Box 516, SE-75120 Uppsala, Sweden; 2 Institute Laue Langevin (ILL), 71 Avenue des Martyrs, 38000 Grenoble, France; 3 Center for Nanophase Materials Sciences (CNMS), Oak Ridge National Laboratory, 1 Bethel Valley Road, TN 37831 Oak Ridge, USA; 4 Juelich Centre for Neutron Science, Forschungszentrum Juelich GmbH Outstation at SNS, 1 Bethel Valley Road, Oak Ridge, TN 37831 USA

## Abstract

We present *in-situ* neutron spin echo measurements on an entangled polydimethylsiloxane melt under shear and demonstrate the ability to monitor nano-scale dynamics in flowing liquids. We report no changes in the topological interactions of the chains for shear rates approaching the inverse longest relaxation time. Further experiments following along this line will allow to systematically test the predictions of theories, like e.g. convective constraint release.

## Introduction

The unique flow properties of non-Newtonian materials result from relaxation processes occurring on time scales relevant for our daily lives and relate to the temporal evolution of topological interactions. How these affect the macroscopic flow properties is still a subject of intense discussion.

In the early 1970’s de Gennes, Doi and Edwards described the dynamics of a polymer chain by the reptation model^1–3^, assuming a tube-like confining field restricting the motion of a single chain. Later, the mechanism of contour length fluctuations (CLF)^4^ was added, including the fluctuations of the path length of occupied tube segments. While the reptation model is exceptionally successful in explaining the viscoelastic properties of polymeric fluids and predicting scaling laws, some experimental observations were initially not well captured. In order to overcome this challenge several modifications were proposed. Constraint release (CR) takes the dynamics of the entanglements into account and suggests an additional, Rouse-like relaxation mechanism of the tube^5,6^. For shear rates exceeding the inverse reptation time ($\dot{\gamma }\ge \frac{1}{{\tau }_{{\rm{D}}}}$, with $\dot{\gamma }$ and *τ*_D_ the shear rate and the tube relaxation time, respectively), the convection resulting from steady flow renews the entanglements of any chain and defines the relaxation of the tube surroundings^7,8^. This effect is called convective constraint release (CCR) and was combined with CLF by Likhtman *et al*.^9,10^. Further refinements of the CCR model consider convective conformational renewal describing the loss of orientation of the unstretched chain due to flow-induced elongation of the tube segments^11^. For high shear rates ($\dot{\gamma }\ge \frac{1}{{\tau }_{{\rm{r}}}}$, with *τ*_r_ the Rouse time) chain stretch is considered in the Doi-Edwards-Marrucci-Grizzuti (DEMG) theory^12,13^. For a detailed overview of the above mentioned theories as well as their experimental verifications we refer to the review by McLeish^14^.

More recently, the GLaMM model combining reptation, CCR and chain stretch, as well as approximatively CLF, was developed and provides mechanical stresses and single chain structure factors in good agreement with experiments^9^. Still the theory faces challenges in predicting the correct normal stress values for high shear rates $\dot{\gamma }{\tau }_{r} > 15$, which might be related to the underestimation of chain retraction. Slip-link^15,16^, primitive chain network model based^17^ as well as non equilibrium molecular dynamics simulations^18,19^ and particle dynamics simulations^20^ suggested a shear-induced decrease in entanglement density, which was not captured by the GLaMM model. These findings were included in a revision of the CCR model^21,22^.

On the experimental side, small angle neutron scattering (SANS) studies provide structural information on the chain conformation^23,24^ and quasielastic scattering can provide some insights into dynamics^25^ under shear. Neutron spin echo spectroscopy (NSE) is a powerful tool for the investigation of slower dynamics of polymers^26^ and allowed to verify the predictions of the reptation model^27–29^, CLF^30^ and thermal CR^31^, but a proper experimental protocol that can monitor simultaneously the shear-induced microscopic chain confinement and the space-time evolution of the dynamic response, is yet to be established.

In this work we investigate the field constraining the motion of the individual chain by successfully applying high resolution NSE to entangled polymers under shear. We present SANS and NSE data on a high molecular mass polydimethylsiloxane (PDMS) melt at the onset of the shear thinning $(\dot{\gamma }\approx \frac{1}{{\tau }_{D}})$, where CCR might contribute to the relaxation mechanism. In analogy to rheo-SANS, in the following the terminology rheo-NSE is adopted for neutron spin echo measurements conducted under shear. Our results suggest that the topological interactions probed by the intermediate scattering function remain unchanged.

## Results

**Figure 1:**
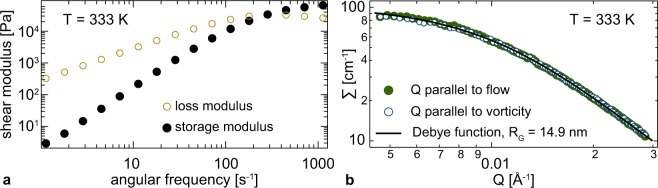
PDMS melt characterization. Panel (a): Storage and loss moduli of the 192 kg/mol (fully protonated) PDMS plotted over frequency and measured in cone-plate geometry. Panel (b): Macroscopic scattering cross section extracted from SANS data plotted versus Q.

The PDMS was synthesised by anionic ring-opening polymerization of D3, following the specific methods described by Bellas, Iatrou, and Hadjichristidis^32–35^. For the neutron measurements a melt containing 62.6% fully deuterated chains of molecular weight *M*_*w*_ = 191 kg/mol (PDI = 1.08), and 37.4% fully protonated chains of *M*_*w*_ 192 kg/mol (PDI = 1.08) was prepared, by solving and mixing in toluene with subsequent evaporation. Assuming a molecular weight between two entanglements of *M*_*e*_ = 12 kg/mol^36^, corresponding to roughly 30 Kuhn segments, this results in 16 entanglements. The longest relaxation time *τ*_t_ = 4 ms was determined in cone-plate geometry on an Anton Paar MCR 501 rheometer (panel a) of Fig. 1). Considering the 16 entanglements, we extract *τ*_r_ = 0.25 ms. In addition, a series of PDMS melts has been measured in contact with polished silicon surfaces in plate-plate geometry^37^. The slip length was estimated by varying the distance between the two plates and never exceeded 20 *μ*m, being negligible given the gaps on the mm range in the neutron shear cell. Moreover, combinations of different pairs of plate distances produced the same result within 10 *μ*m showing that shear banding is unlikely to happen in our set-up. Note, both surface slip and shear banding are macroscopic effects, strongly influencing and limiting rheological studies. NSE, however, is sensitive to the dynamics on the nm length scale. Changes in the topological interactions can be detected independent whether slip or banding is present as long as the local shear rate over the tube diameter is large enough. However, the local shear rate is affected by the above mentioned effects. The same argument applies to other effects that make rheological studies of polymer melts challenging, like e.g. edge fracture.

The PDMS melt was studied at rest and at a shear rate of 100 s^−1^. At this shear rate, no structural anisotropy was found in a SANS investigation, probing a Q-range from 0.03 to 0.4 nm^−1^ (Fig. 1, panel b)). The fitted single chain radius of gyration is 149.4(4) Å and 149.2(4) Å in flow and vorticity direction, respectively. At the present shear rates chain stretching is not expected^12,13^.

**Figure 2:**
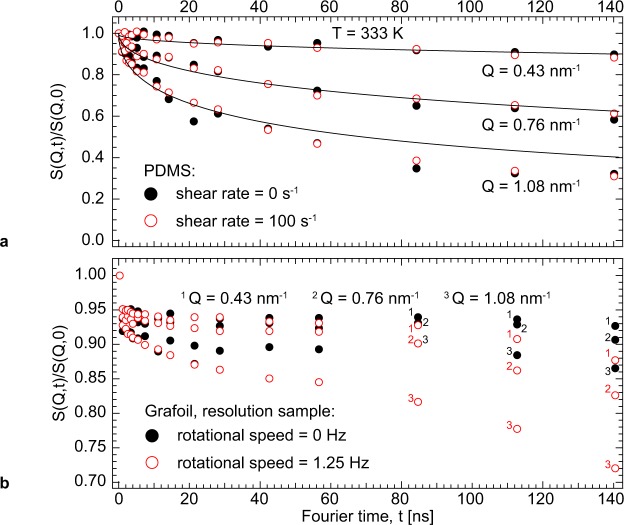
Rheo-NSE and resolution measurements. Panel (a): S(Q, t)/S(Q, 0) of the PDMS melt measured without shear (closed symbols) and at shear rate 100 s^−1^ (open symbols, 1.25 Hz cone rotational speed). Solid black lines represent the fit to reptation dynamics. Panel (b): S(Q, t)/S(Q, 0) resolution functions measured on tightly wound grafoil without rotation (closed symbols) and rotating at 1.25 Hz (open symbols), used for the reduction of the PDMS data. In both panels (a and b), the statistical errors on the single measurement points do not exceed the plot marker radius.

In order to extract the tube diameter d and relaxation time *τ*_D_, we conducted a simultaneous fit of the whole NSE data set taken without shear (Fig. 2) to the reptation model (1)^28^.

1 $$\frac{{S}_{\mathrm{coh},\mathrm{LocRep}}(Q,t)}{{S}_{\mathrm{coh},\mathrm{LocRep}}(Q,\mathrm{0)}}={S}_{{\rm{loc}}}(1-{e}^{-{(\frac{Qd}{6})}^{2}})+{S}_{{\rm{rep}}}{e}^{-{(\frac{Qd}{6})}^{2}}$$

The model distinguishes between the contribution of local chain fluctuations inside the confining field (2), where *τ*_0_ is the characteristic time scale, and the contribution of the reptational diffusion of the chain out of the confinement (3), where *τ*_D_ is the characteristic time scale, with ${\tau }_{0}=\frac{36}{W{l}^{4}{Q}^{4}}$ and ${\tau }_{{\rm{D}}}=\frac{3{N}^{3}{l}^{2}}{{\pi }^{2}W{d}^{2}}$, respectively.

2 $${S}_{{\rm{loc}}}={e}^{\frac{t}{{\tau }_{0}}}{\rm{erfc}}\sqrt{\frac{t}{{\tau }_{0}}}$$

3 $${S}_{{\rm{rep}}}=\frac{8}{{\pi }^{2}}{\sum }_{{\rm{n}}={\rm{odd}}}\frac{1}{{n}^{2}}{e}^{-\frac{{n}^{2}t}{{\tau }_{{\rm{D}}}}}$$

The fit (Fig. 2, solid lines) corresponds to a tube diameter d = 12.335(4) nm, a *τ*_0_*Q*^4^ = 162.25(3) ns/nm^4^, and a reptation time *τ*_D_ = 4.2(7) ms, which is in very good agreement with *τ*_t_. In this fit d, *τ*_0_*Q*^4^ and *τ*_D_ were unconstrained fitting variables. The fitting error estimates are calculated only from the statistical error. Determining *τ*_D_ from the fitted tube diameter and by fixing the Rouse frequency W to W = 6.84 ns^−1^ (calculated from temperature dependency following ref.^38^), the segment length to l = 0.63 nm (according to ref.^26^) and the number of chain segments N = (chain mass)/(monomer mass) = 2589, yields *τ*_D_ = 2 ms, which is in good agreement both with *τ*_t_ and the value for *τ*_D_ obtained as free fitting parameter. The single chain dynamics measured under shear at the shear-thinning onset show no deviation from the zero-shear dynamics (Fig. 2a).

**Figure 3:**
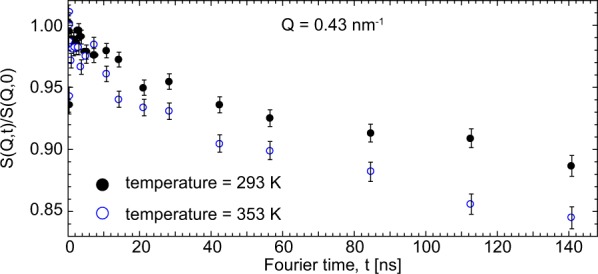
NSE measurements conducted without shear at different temperatures. S(Q, t)/S(Q, 0) plotted over Fourier time for temperatures of 293 K and 353 K taken at Q = 0.43 nm^−1^. Error bars denote the statistical error.

To verify the absence of shear-driven degradation of the sample material, zero-shear measurements had been performed both before and after the measurements at $\dot{\gamma }=100$ s^−1^. No deviation of statistical significance was observed. Finally, also the effect of temperature on the intermediate scattering function was investigated (Fig. 3). Higher temperatures, which could result from friction, manifest themselves in a sharp drop of S(Q, t)/S(Q, 0) at the lowest Fourier times and an offset of the plateau at elevated Fourier times. The relaxation mechanisms characterized by *τ*_0_ and *τ*_D_ both depend on temperature through the Rouse frequency $W=\frac{3{k}_{B}T{l}^{2}}{{\zeta }_{0}}$ (*ζ*_0_ being the polymer segment friction coefficient), thus the model predicts an acceleration of both relaxation modes as temperature increases. This leads to an increasing offset of the plateau in the model intermediate scattering function, which here is experimentally confirmed by the measurement. No changes in chain dynamics due to local friction-induced heating are observed in the measurements conducted under shear.

## Discussion

In the present study, the measured zero-shear dynamics deviate at large length and time scales from the intermediate scattering function predicted by the reptation model (Fig. 2a, Q = 1.08 nm^−1^, t > 40 ns). We note that the fitting model used does not take into account CLF and CR. The present experimental design centres on the effect of shear and is ill fit for a detailed analysis of the discrepancy between zero-shear dynamics and the model prediction. However, past systematic NSE studies dedicated separately to CLF and CR indicate that CLF affects dynamics both at low and high Q values^30^, while the deviation due to thermal CR is most pronounced at high Q^31^.

For shear rates exceeding the inverse *τ*_D_, the theories of convective constraint release^7^ consider a shear-induced convection in the tube-like confining field allowing a Rouse-like motion of the tube itself ^39,40^. Dissipative particle dynamics simulations^20^ predict chain alignment leading to flow-induced disentanglement and a commensurate tube dilation as well as a broadening of the relaxation time spectrum and non-equilibrium molecular dynamics simulations predict an onset of disentanglement for shear rates approaching inverse *τ*_D_^18,19^. For shear rates of half the inverse of *τ*_D_ we could not detect disentanglement. Still our experiment shows that direct measurement of topological interactions in polymers under shear by means of NSE is possible up to large Fourier times, about three orders of magnitude larger than reported previously^41^, for shear rates approaching the inverse *τ*_D_ of PDMS. Experiments on more rigid polymers, polymers with longer *τ*_D_ or at higher shear rates should reveal possible disentanglement in future studies.

## Methods

**Figure 4:**
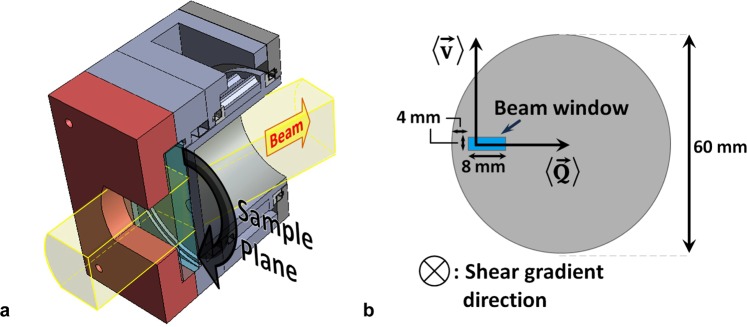
Shear and scattering geometries. Schematics of the cone-plate shear device, panel (a), and scattering geometry, panel (b). The 4 × 8 mm beam window was offset by 4 mm towards the cone center to avoid a scattering contribution from the rotor walls.

Rheo-NSE measurements require shear devices that do not influence the neutron beam polarisation, and the ability to, up to sufficiently high Fourier times, correct for the phase shifts and depolarisation induced by Doppler scattering. To provide steady shear to the polymer melt we designed an air-driven and non-magnetic vertical cone-plate shear cell^41,42^ with a cone angle and radius of 2.86° and 30 mm, respectively (for a schematic see Fig. 4, panel a)). All shown neutron scattering experiments were performed on the instruments D22 and IN15 (both Institut Laue-Langevin, Grenoble), with preliminary NSE measurements performed at SNS-NSE (Oak Ridge National Laboratory). Magnetic stray-fields were monitored and magnetic perturbations (depolarisation less than 2% for a neutron wavelength *λ* = 1.6 nm on IN15) at the sample position can be excluded. The measurements presented here were conducted at *λ* = 1 nm. Further, turning on the pneumatic motor resulted in no measurable additional depolarisation. The scattering experiments were performed with the beam vertically and horizontally collimated to 4 and 8 mm, respectively, and at Q (momentum transfer) values between 0.3 nm^−1^ and 1.1 nm^−1^. $\overrightarrow{Q}$ was oriented perpendicular to the macroscopic flow $< \overrightarrow{v} >$ and velocity gradient direction (see Fig. 4, panel b), the brackets denote mean values). The temperature of the entire cell was 333 K and controlled using a Lauda RP 855 water bath.

The resolution of IN15 for the present setup was determined using a tightly wound grafoil spiral of 3 mm thickness mounted 3 mm behind the cavity for the liquid sample (Fig. 2b). The detector signal was analysed and reduced pixel by pixel in order to account for the Doppler-induced phase differences that arise from a $\overrightarrow{Q}$-vector spread across the detector. In theory, one should calculate the scalar product $(\overrightarrow{Q}\cdot \overrightarrow{v})t$ for each neutron hitting a particular detector pixel, where $\overrightarrow{Q}$ is defined by the detector angle and the pixel position in the detector, $\overrightarrow{v}$ is the local velocity vector of the sample voxel scattered from, and *t* is the Fourier time (a function of magnetic field and neutron drift path); this scalar product defines the Doppler-induced shift in the neutron’s phase angle. In praxis, the Doppler-induced phase shifts must be accounted for statistically through a careful choice of shear geometry, scattering geometry and resolution measurement setup. While the mean of the $(\overrightarrow{Q}\cdot \overrightarrow{v})t$ products of each neutron detected simply shifts the echo phase, a spread of single-neutron phase shifts affects the neutron beam polarisation and diminishes the echo’s amplitude by the factor of $< \,{\cos }(\overrightarrow{Q}\cdot (\overrightarrow{v}- < \overrightarrow{v} > )t) >$. In the present study, the experimental geometry chosen results in a zero mean horizontal sample velocity component but a non-zero mean squared value due to the flow curvature. The shear gradient in the liquid sample has a narrowing effect on the distribution of the horizontal velocity component when combined with strong vertical beam collimation. Hence using a rotating solid resolution for the purpose of data reduction, together with pixel-by-pixel analysis, take here the highest possible beam depolarisation into account. However, the vertical extremes of the detector can suffer from partial depolarisation, because the bulk velocity vectors in the irradiated sample volume are nearly vertical and $\overrightarrow{Q}$ has a vertical component for pixels not positioned on the vertical center line of the detector. To minimize the vertical component of $\overrightarrow{Q}$ in the analysed data, the top and bottom thirds of the detector were omitted for analysis.

## Summary

Our work shows the ability to *in-situ* monitor nano-scale dynamics in flowing liquids with NSE. By this approach macroscopic transitions in flow behaviour, quantifiable e.g. by changes in the storage and loss moduli, can be directly related to their origins at molecular and single-particle scales. Our measurements show no change of the tube diameter and thus no change of the entanglement density for flow rates up to about half the inverse longest relaxation time. This provides an important boundary condition to test molecular theories, especially those including flow-induced disentanglement^22^ and paves the way towards a connection of shear thinning to transitions in the topological interactions between individual polymer chains.
